# The rod signaling pathway in marsupial retinae

**DOI:** 10.1371/journal.pone.0202089

**Published:** 2018-08-29

**Authors:** Nicolas D. Lutz, Emina Lemes, Leah Krubitzer, Shaun P. Collin, Silke Haverkamp, Leo Peichl

**Affiliations:** 1 Max Planck Institute for Brain Research, Frankfurt am Main, Germany; 2 Institute of Medical Psychology and Behavioral Neurobiology, University of Tübingen, Tübingen, Germany; 3 Graduate Training Centre of Neuroscience / IMPRS for Cognitive & Systems Neuroscience, University of Tübingen, Tübingen, Germany; 4 Institute of Cellular and Molecular Anatomy, Dr. Senckenbergische Anatomie, Goethe University Frankfurt, Frankfurt am Main, Germany; 5 Center for Neuroscience, University of California Davis, Davis, California, United States of America; 6 The Oceans Institute and Oceans Graduate School, The University of Western Australia, Crawley, Western Australia, Australia; Doheny Eye Institute/UCLA, UNITED STATES

## Abstract

The retinal rod pathway, featuring dedicated rod bipolar cells (RBCs) and AII amacrine cells, has been intensely studied in placental mammals. Here, we analyzed the rod pathway in a nocturnal marsupial, the South American opossum *Monodelphis domestica* to elucidate whether marsupials have a similar rod pathway. The retina was dominated by rods with densities of 338,000–413,000/mm². Immunohistochemistry for the RBC-specific marker protein kinase Cα (PKCα) and the AII cell marker calretinin revealed the presence of both cell types with their typical morphology. This is the first demonstration of RBCs in a marsupial and of the integration of RBCs and AII cells in the rod signaling pathway. Electron microscopy showed invaginating synaptic contacts of the PKCα-immunoreactive bipolar cells with rods; light microscopic co-immunolabeling for the synaptic ribbon marker CtBP2 confirmed dominant rod contacts. The RBC axon terminals were mostly located in the innermost stratum S5 of the inner plexiform layer (IPL), but had additional side branches and synaptic varicosities in strata S3 and S4, with S3-S5 belonging to the presumed functional ON sublayer of the IPL, as shown by immunolabeling for the ON bipolar cell marker Gγ13. Triple-immunolabeling for PKCα, calretinin and CtBP2 demonstrated RBC synapses onto AII cells. These features conform to the pattern seen in placental mammals, indicating a basically similar rod pathway in *M*. *domestica*. The density range of RBCs was 9,900–16,600/mm^2^, that of AII cells was 1,500–3,260/mm^2^. The numerical convergence (density ratio) of 146–156 rods to 4.7–6.0 RBCs to 1 AII cell is within the broad range found among placental mammals. For comparison, we collected data for the Australian nocturnal dunnart *Sminthopsis crassicaudata*, and found it to be similar to *M*. *domestica*, with rod-contacting PKCα-immunoreactive bipolar cells that had axon terminals also stratifying in IPL strata S3-S5.

## Introduction

Vision in low light (scotopic) conditions depends on the retinal signaling pathways of the rod photoreceptors. The basic retinal circuitry for scotopic vision is well understood in placental mammals [1,2], whereas there is little data on the rod pathway in another major clade of mammals, the marsupials. Marsupials form a major group of mammals whose ancestors radiated early in evolution. Thus, they can provide some insight into the structure and function of the retina in early mammals, and how the retina evolved in different lineages with alterations in lifestyle. Here we focus on a nocturnal marsupial, the South American gray short-tailed opossum *Monodelphis domestica*, which has been introduced as a model species for developmental and comparative research [3].

A hallmark of the retinae of nocturnal placental mammals is the dominance of rod photoreceptors compared to cone photoreceptors [4]. This is also true for the nocturnal marsupials that have been investigated [5–8]. Although the proportions of cones in marsupials are low, these and other studies found evidence for cone-based di- or even trichromatic color vision in some marsupials [7–12]. In placental mammals, the retinal pathways for diurnal cone signaling and nocturnal rod signaling are largely separated [1]. The rod photoreceptors transmit light-induced signals via specialized ribbon synapses from their axon terminals, the rod spherules, to rod bipolar cells (RBCs), which comprise only a single type of bipolar cell compared to about a dozen types of cone bipolar cells [2,13]. As RBCs are depolarized by light stimuli and their axons terminate in the innermost part of the inner plexiform layer (IPL) [14], they are considered to be strictly ON bipolar cells. In the ‘classical’ rod pathway, RBCs send their signals via sign-conserving ribbon synapses to a specific type of amacrine cell, the AII cells, which, in turn, transmit signals via sign-conserving gap junctions to ON cone bipolar cells, and via sign-inverting inhibitory chemical synapses to OFF cone bipolar cells, which finally contact ON and OFF ganglion cells [1,15]. Not much is known about the corresponding cell types in marsupial retinae. Here, we focus on RBCs and the rod signaling pathway of the *M*. *domestica* retina and present some comparative data for the Australian nocturnal fat-tailed dunnart *Sminthopsis crassicaudata*.

## Materials and methods

### Ethics statement

All procedures for *Monodelphis domestica* husbandry, breeding and euthanasia complied with the National Institutes of Health Principles of Laboratory Animal Care and were approved by the Institutional Animal Care and Use Committee of the University of California, Davis, CA, USA (permit number 20347). *Sminthopsis crassicaudata* tissue was obtained from an animal euthanized in a study that complied with the Australian Government’s code for the care and use of animals for scientific purposes, and was approved by the Institutional Animal Ethics Committee of The University of Western Australia, Crawley, WA, Australia (permit number 03/100/1123).

### Animals and tissue preparation

Eyes were obtained from reproductively mature (6–10 months old) gray short-tailed opossums (*Monodelphis domestica*, Didelphidae) at the breeding colony at the University of California, Davis (Davis, CA, USA). Animals were euthanized with an overdose of sodium pentobarbital (Beuthanasia; >250 mg/kg, IP) and transcardially perfused with phosphate buffered saline (PBS) followed by 4% paraformaldehyde (PFA) in 0.1 M PBS (pH 7.4) for 30 min. Following perfusion, the eyes were enucleated. For immunofluorescence labeling, the eyes were postfixed in 4% PFA for 1.5 h. For electron microscopy, the eye was postfixed in 4% PFA for 1 h for pre-embedding immunohistochemical staining, and for 24 h for transmission electron microscopy alone. For comparison, one retina of an adult fat-tailed dunnart (*Sminthopsis crassicaudata*, Dasyuridae) was obtained when the animal was euthanized for an unrelated study at the School of Biological Sciences, The University of Western Australia. This eye was immersion-fixed overnight in 4% PFA.

After rinsing in PBS, the eyes were transferred to PBS with 0.1% NaN_3_ for preservation during storage. For longer storage, eyes were cryoprotected by successive immersion in 10%, 20% and 30% (w/v) sucrose in phosphate buffer (PB), and frozen at -20°C. Subsequently, eyes were thawed and retinae were isolated from the vitreous body, choroid and sclera and further processed.

For frozen vertical sections of the retina (i.e., perpendicular to the retinal layers) the tissue was transferred from 30% sucrose to tissue freezing medium (Leica Biosystems, Wetzlar, Germany), frozen, sectioned at 16 μm thickness with a cryostat (Leica CM 3050 S, Wetzlar, Germany), and collected on Superfrost Plus slides (Menzel Gläser, Braunschweig, Germany). For thick sections, retinae were embedded in 4% agar, and 60 μm thick slices were cut with a vibratome (Leica VT1000 S, Wetzlar, Germany).

### Immunohistochemistry

To identify photoreceptor types, we used the mouse monoclonal antibody rho4D2 to detect rod opsin (dilution 1:500; kindly provided by R. Molday [16]), the rabbit antiserum JH 492 to label the middle-to-longwave-sensitive (LWS) cone opsin (dilution 1:2000; kindly provided by J. Nathans [17]), and the goat antiserum sc-14363 to label the shortwave-sensitive (SWS1) cone opsin (dilution 1:500; Santa Cruz Biotechnology Inc., Santa Cruz, CA, USA). For the detection of RBCs, we used a rabbit anti-protein kinase Cα (PKCα) antiserum (dilution 1:5000 for fluorescence microscopy and 1:2500 for electron microscopy; Cat.-No. P4334, Sigma-Aldrich, Munich, Germany), which has been shown to specifically label RBCs in a broad range of mammalian species [18–22]. In some cases, a goat anti-PKCα antiserum was used (dilution 1:1000; Cat.-No. sc-208-G; Santa Cruz). A goat antiserum against choline acetyltransferase (ChAT; dilution 1:200; Cat.-No. AB144P, EMD Millipore, Darmstadt, Germany) was used to detect the outer and inner ChAT bands belonging to the OFF and ON sublayers of the IPL, respectively. ON bipolar cells were labeled with a rabbit antiserum against G-protein γ13 (Gγ13) (dilution 1:1000; kindly provided by R. F. Margolskee [23]). As a marker for AII cells, we used a goat antiserum against calretinin (dilution 1:2000; AB1550, EMD Millipore) [24–26], and as a marker for synaptic ribbons, we used a mouse antibody against C-terminal binding protein 2 (CtBP2; dilution 1:5000; Cat.-No. 612044, BD Biosciences, Heidelberg, Germany) [27]. Secondary fluorophore-conjugated antibodies were used to detect primary antibodies by indirect immunofluorescence. Specifically, we used various combinations of Alexa 488-conjugated, Cy3-conjugated and Cy5-conjugated donkey anti-rabbit, donkey anti-goat and donkey anti-mouse IgG antibodies. Immunohistochemistry for fluorescence microscopy and transmission electron microscopy was performed following established protocols [28–30].

For fluorescence microscopy, cryostat sections were washed in PB for 10 min and preincubated for 1 h in PB with 0.5% Triton X-100 and 10% normal goat serum or normal donkey serum. The sections were then incubated overnight in primary antibody solution containing 0.5% Triton X-100 and 3% normal goat serum or normal donkey serum. After rinsing in PB, they were incubated in secondary antibody solution for 1 h. For some sections, the secondary antibody solution also contained 4,6-diamidino-2-phenylindole (DAPI) as a fluorescent nuclear stain to reveal the general retinal layering. After rinsing in PB again, sections were coverslipped with Aqua Poly/Mount (Polysciences Inc., Warrington, PA, USA). Two whole retinae were triple-immunolabeled free-floating with the PKCα, calretinin and CtBP2 antibodies following the above protocol, but with an incubation time of three days in the primary antibody solution. For double and triple immunolabeling, the incubation solutions contained a mixture of the primary antibodies and appropriate secondary antibodies, respectively.

For transmission electron microscopy, vibratome slices were used. For detailed analysis of the ultrastructure, the slices were immediately post-fixed with 0.5% OsO_4_, dehydrated and embedded in epoxy embedding medium (EPON). For pre-embedding immunostaining, free-floating vibratome slices were incubated in 10% normal goat serum for 2 h and then in primary antibody solution (rabbit anti-PKCα) with 3% normal goat serum, 0.05% NaN_3_ and 0.02% Triton-X-100 for three days at 4°C. After rinsing in PBS, the slices were incubated in goat anti-rabbit IgG (1:100; Vector, Burlingame, CA) overnight, before they were incubated with rabbit peroxidase-anti-peroxidase antiserum (PAP; dilution 1:100; Cat.-No. P1291, Sigma-Aldrich) for 12 h at 4°C. The slices were rinsed again in PBS, subsequently in 0.05 M Tris-HCl, pH 7.6, and then treated with 3,3-diaminobenzidine (DAB; 0.05% in Tris-HCl) with 0.01% H_2_O_2_ for 5–10 min. After rinses in Tris-HCl and subsequently in 0.1 M cacodylate buffer (pH 7.4), the slices were postfixed in 2.5% glutaraldehyde in cacodylate buffer for 1 h. After several washes in distilled water, the DAB reaction product was silver-intensified by incubating the slices in a solution containing 2.6% hexamethylenetetramine, 0.2% silver nitrate and 0.2% disodium tetraborate for 10 min at 60°C. The sections were then rinsed in distilled water and treated for 3 min with 0.05% gold chloride, rinsed again and incubated for 3 min in 2.5% sodium thiosulfate. Subsequently, the sections were postfixed with 0.5% OsO_4_ in cacodylate buffer for 30 min and dehydrated in a graded series of acetones. They were embedded in Epon and serial ultrathin (60 nm) sections were cut and stained with uranyl acetate.

### Imaging and analysis

Some immunofluorescence-labeled tissue was analyzed with a Zeiss Axio Imager.Z1 ApoTome fluorescence microscope, equipped with an oscillating grating in the epifluorescence beam. Images were taken as stacks of several optical sections, using a cooled charge-coupled device (CCD) camera, an EC Plan-Neofluar 40x/0.75 M27 objective and the Zeiss Axiovision LE software. Some immunofluorescence-labeled tissue was analyzed using a Zeiss LSM 710 microscope and Zen 2009 software. Confocal image stacks were taken at z-distances between 0.46 and 1.22 μm, using different objectives (LD LCI Plan-Apochromat 25x/0.8 Imm Corr DIC M27, C-Apochromat 40x/1.2 W Corr M27). Images were viewed and projections were examined with ImageJ (http://imagej.nih.gov/ij/); cells were counted using the cell counter plugin. Images for illustration were adjusted for brightness and contrast. Electron microscopic images were taken with a Zeiss Leo912 AB Omega transmission electron microscope and photographed with a wide-angle Dual Speed 2K-CCD camera using the ImageSP software (TRS, Moorenweis, Germany).

*Monodelphis domestica* RBC and AII cell densities were assessed in 26 and 25 sample fields across the two immunostained wholemounts md1 and md2, respectively. Each field was imaged from the inner plexiform layer to the outer plexiform layer, and cells were counted by focusing through the stack. Counting was done by three independent observers, and the inter-rater agreement was very high, with 93.9 ± 3.5% for AII cells and 96.2 ± 2.4% for RBCs (means ± SD). The sample fields were the same for both cell types, so that local RBC/AII ratios could be determined directly. Counting field sizes were 354 x 354 μm; in some cases, RBCs were counted in smaller subfields. Rod densities could be assessed in some regions of one retina with differential interference contrast (DIC) in small sampling fields of between 10 x 15 μm and 20 x 20 μm.

## Results

### Photoreceptors

**Fig 1:**
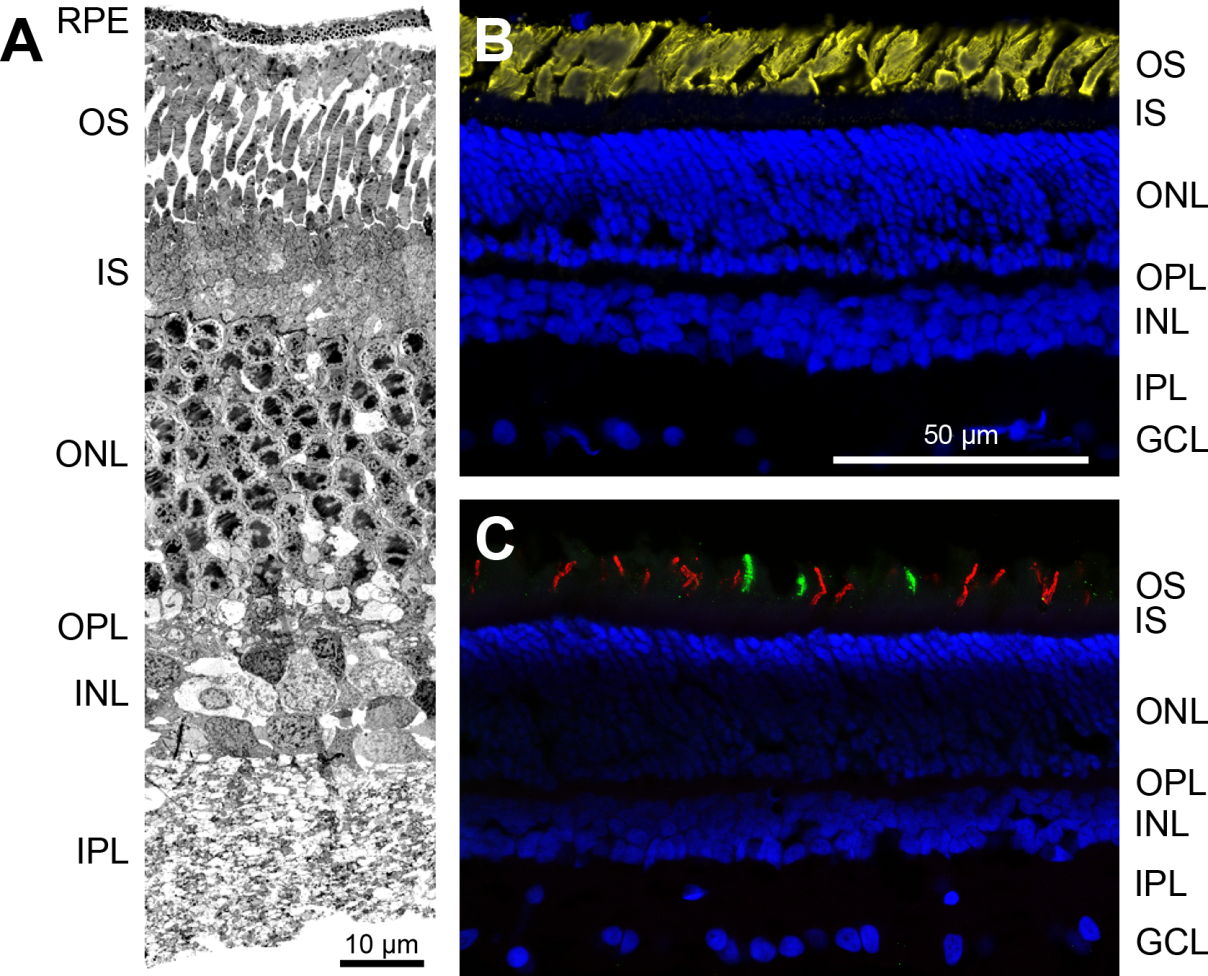
Transverse sections of the *Monodelphis domestica* retina. (**A**) The electron micrograph of an ultrathin transverse section shows a typical retinal layering as seen in nocturnal placental mammals. The thickest layer is the outer nuclear layer (ONL), containing the photoreceptor somata. (**B**) Immunolabeling of a transverse cryostat section for rod opsin (yellow) shows the densely packed rod outer segments; counterstaining with DAPI (blue) shows the retinal layers. (**C**) Double-immunolabeling of a transverse cryostat section for shortwave-sensitive SWS1 (green) and middle-to-longwave-sensitive LWS cone opsin (red) shows the opsin-containing cone outer segments of the sparse cone populations; counterstaining with DAPI (blue). Images in (B) and (C) are maximum intensity projections of confocal image stacks. RPE, retinal pigment epithelium; OS, photoreceptor outer segments; IS, photoreceptor inner segments; OPL, outer plexiform layer; INL, inner nuclear layer; IPL, inner plexiform layer, GCL, ganglion cell layer. Scale bar in (**B**) applies to (B, C).

Electron micrographs of transverse sections of the *M*. *domestica* retina revealed the typical layering seen in nocturnal placental mammals (Fig 1A). Retinal thickness was about 125 μm; the thickest layer was the outer nuclear layer (ONL) with approximately eight tiers of photoreceptor somata. Most of these somata had nuclei with large dark heterochromatin aggregations reminiscent of coffee beans, indicating the inverted nuclear architecture typical for the rods of nocturnal placental mammals [31]. Immunostaining for rod opsin confirmed a high rod density (Fig 1B; for numbers, see Results section ‘Densities of rods, rod bipolar cells and AII amacrine cells’). Our stained sections further revealed a considerable number of cones expressing the middle-to-longwave-sensitive (LWS) cone opsin and a smaller number of cones expressing the shortwave-sensitive (SWS1) cone opsin (Fig 1C). We observed no ‘dual pigment’ cones expressing both opsins.

### Rod bipolar cells

**Fig 2:**
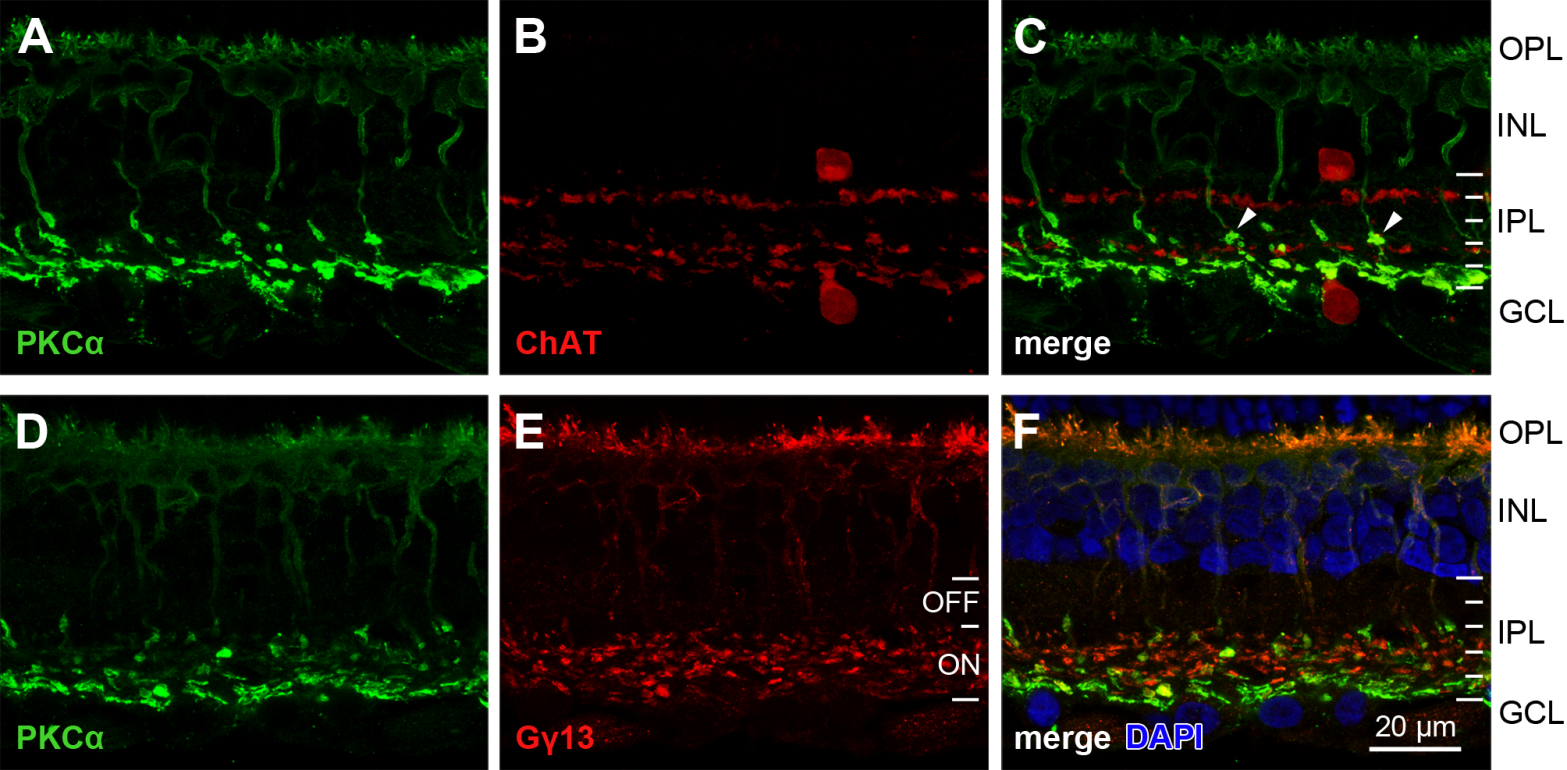
PKCα-ir bipolar cells in *M*. *domestica*. (**A-C**) Double-immunolabeling for PKCα and ChAT. (**A**) PKCα immunostaining labels bipolar cells with dendrites in the OPL, somata in the INL and thick axon terminations in the IPL. (**B**) ChAT immunostaining labels the two populations of cholinergic amacrine cells, one with somata in the INL and dendrites stratifying at the boundary between strata S1 and S2 of the IPL, and one with somata in the GCL and dendrites stratifying in S4. (**C**) The merged image of the two labels localizes the majority of PKCα-ir axon terminals to stratum S5, but there also are some side-branches and *en passant* varicosities above the inner ChAT band (two indicated by arrowheads). (**D-F**) Double-immunolabeling for PKCα and the general ON bipolar cell marker Gγ13. (**D**) Same bipolar cell morphologies as in (A). (**E**) The Gγ13 label shows that presumed ON bipolar cell axon terminals occupy strata S3-S5 of the IPL. (**F**) The merged image shows that the axon terminals and varicosities of the PKCα-ir presumed RBCs occupy S3-S5, characterizing them as presumed ON cells. Counterstaining with DAPI (blue) localizes the PKCα-ir somata to the outer part of the INL. The white IPL tick marks in (C, F) divide the IPL into five strata S1 (top) to S5 (bottom) of equal width, the white tick marks in (E) divide the IPL into the presumed functional OFF (S1-S2) and ON (S3-S5) sublayers. Abbreviations as in Fig 1. Images are maximum intensity projections of confocal image stacks. The inner ChAT band in (B, C) appears broader than the outer one, and there are apparent ChAT-ir structures that also are PKCα-ir. Some of this may be a projection artifact, because single-labeled structures that are located behind each other in the z-axis appear to be double-labeled in the projection plane. Some of the apparent double-label is bleedthrough between the fluorescence filters, because it is strongest in the most intensely PKCα-ir elements. Scale bar applies to (A-F).

PKCα immunostaining of *M*. *domestica* retinal sections showed specific labeling of somata in the inner nuclear layer (INL), with dendrites in the outer plexiform layer (OPL) and axons terminating in the inner plexiform layer (IPL), i.e., the typical morphology of bipolar cells (Fig 2). Counterstaining with the nuclear stain DAPI (Fig 2F) showed a localization of the PKCα-immunoreactive (PKCα-ir) somata in the outermost part of the INL. Co-immunostaining for cholinergic amacrine cells (antiserum against choline acetyltransferase, ChAT) showed that the PKCα-ir axon terminals were localized in the inner sublayer of the IPL, mostly below the inner of the two ChAT bands (Fig 2B and 2C), which is typical for RBCs in placental mammals. Interestingly, we also found short side-branches and *en passant* varicosities on PKCα-ir axons above the inner ChAT band, suggesting the synaptic output of the PKCα-ir cells to be bistratified or broadly stratified. By convention, the IPL is divided into five equal strata S1 (near INL) to S5 (near GCL). The *M*. *domestica* ChAT bands were located at the S1/S2 boundary and in S4. Immunostaining of the IPL using Gγ13 antiserum, a general ON bipolar cell marker in placental mammals [23], showed that the presumed functional ON sublayer occupied the inner strata S3 to S5 in *M*. *domestica*, and hence the presumed functional OFF sublayer occupied S1 and S2 (Fig 2E and 2F). PKCα-ir axon terminals and varicosities were restricted to strata S3-S5 (Fig 2F), indicating that despite their broad stratification, the PKCα-ir bipolar cells presumably are functional ON cells.

**Fig 3:**
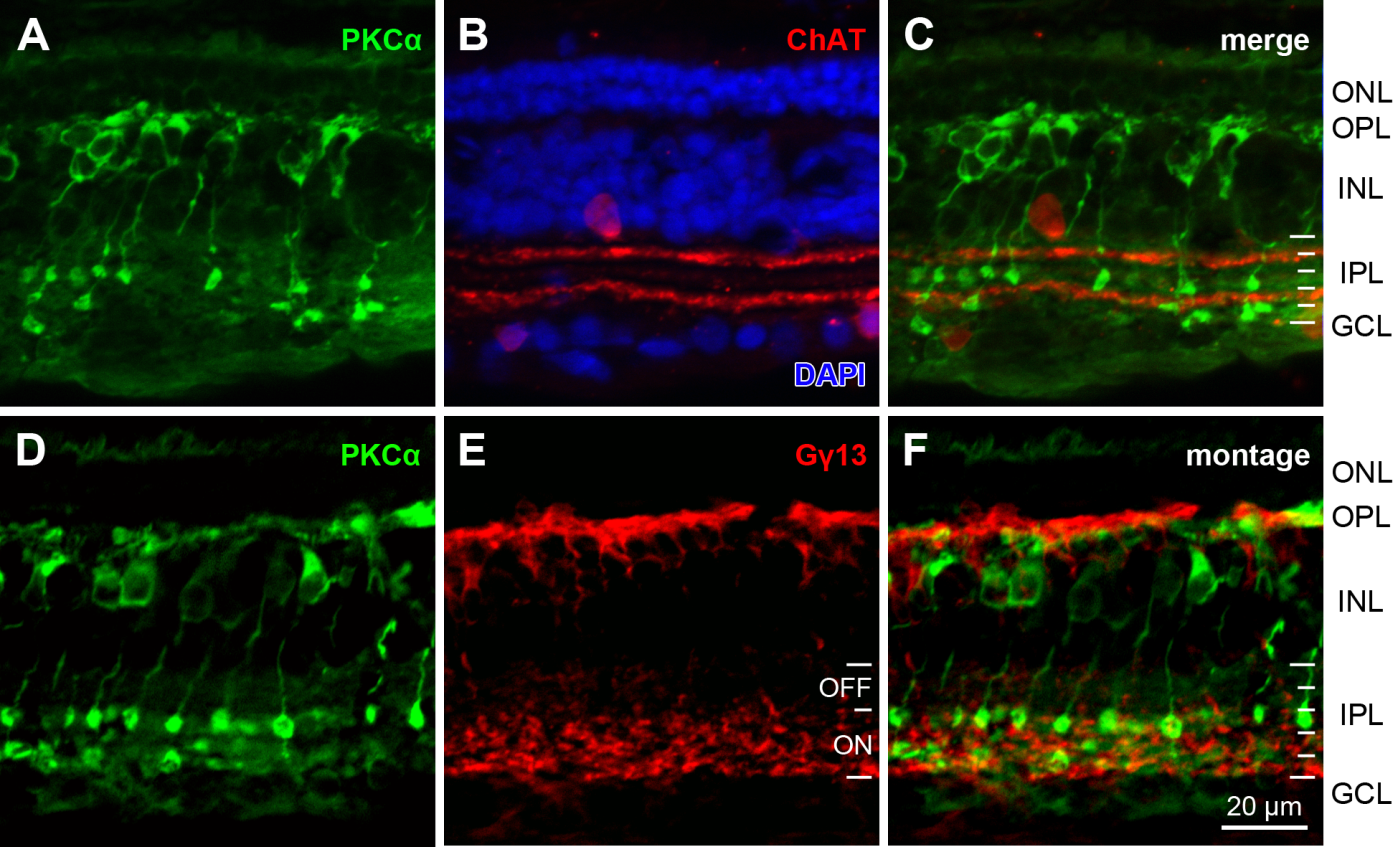
PKCα-ir bipolar cells in *Sminthopsis crassicaudata*. (**A-C**) Double-immunolabeling for PKCα and ChAT. (**A**) PKCα immunostaining labels bipolar cells with dendrites in the OPL, somata in the INL and globular axon terminations in the IPL. The axon terminals and varicosities form at least two bands in the IPL. (**B**) ChAT labeling of the two cholinergic amacrine cell populations, one with somata in the INL and dendrites stratifying at the S1/S2 boundary of the IPL, and one with somata in the GCL and dendrites stratifying in S4. Counterstaining with DAPI (blue) shows the retinal layers and localizes the PKCα-ir somata to the outer part of the INL. (**C**) The merged image of the two labels localizes the PKCα-ir axon terminals and varicosities below and above the inner ChAT band. (**D-F**) Immunolabeling for PKCα and the general ON bipolar cell marker Gγ13. The antisera used here were both from rabbit, hence double-immunolabeling was not feasible and single-labeling was performed on equivalent sections. (**D**) Same bipolar cell morphologies as in (A). (**E**) Gγ13 label shows that presumed ON bipolar cell axon terminals occupy strata S3-S5 of the IPL. (**F**) Overlay (montage) of (D, E) shows that the axon terminals and varicosities of the PKCα-ir presumed RBCs also occupy S3-S5, characterizing them as presumed ON cells. White IPL tick marks as in Fig 2, abbreviations as in Fig 1. Images are maximum intensity projections of confocal image stacks. Scale bar applies to (A-F).

Similar labeling in retinal sections of the Australian marsupial *Sminthopsis crassicaudata* also revealed PKCα-ir bipolar cells with dendrites in the OPL, somata in the outer part of the INL, and globular axon terminations in the inner part of the IPL (Fig 3). The axon terminals and varicosities formed at least two bands in the IPL, one below and one above the inner ChAT band, but all terminals were restricted to strata S3-S5 that the Gγ13 label identified as presumed ON strata. In summary, the PKCα-ir bipolar cells of both *M*. *domestica* and *S*. *crassicaudata* resemble the typical morphology of rod bipolar cells seen in placental mammals, with the exception of a broader axon terminal stratification in the IPL.

### Rod bipolar cell connections

**Fig 4:**
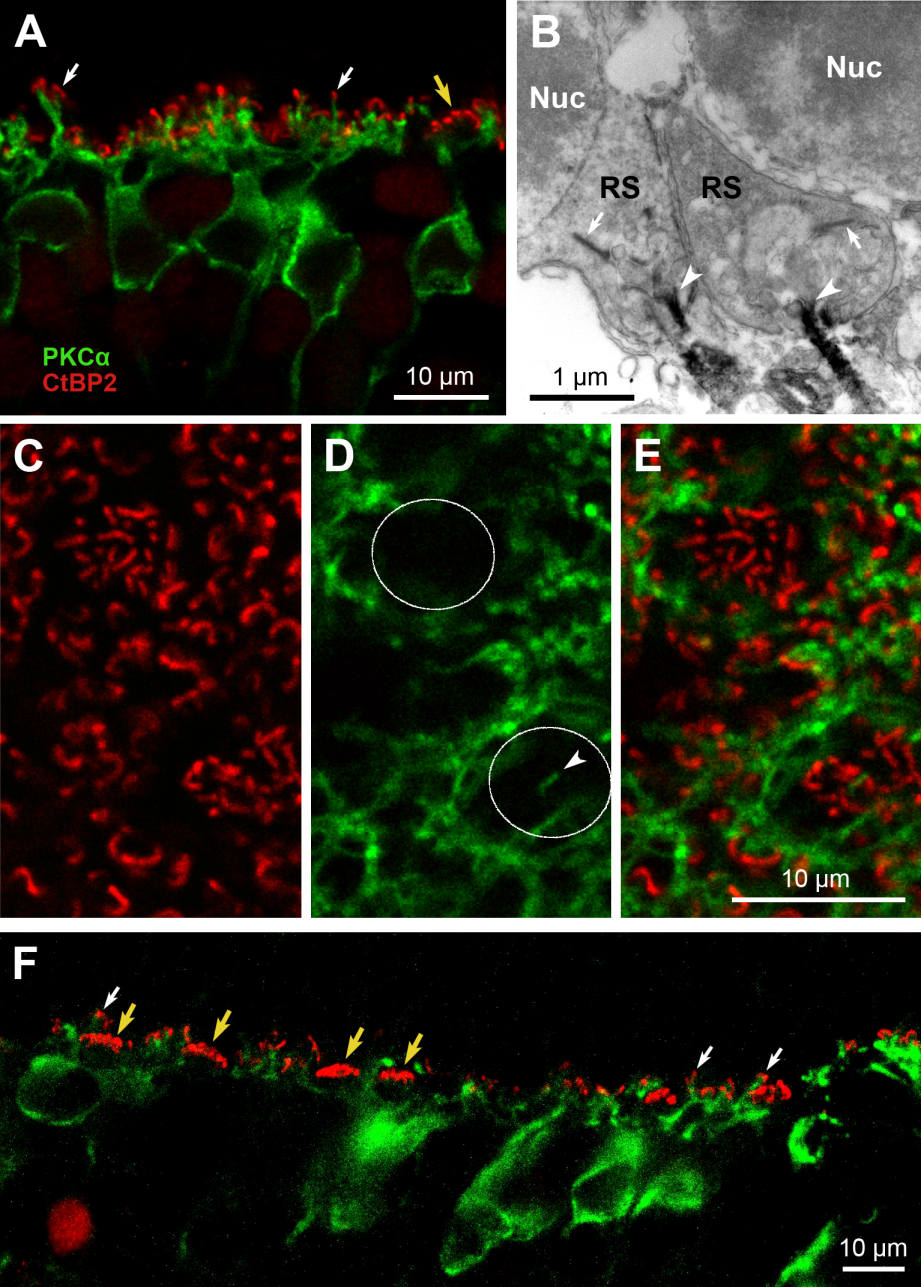
**Synaptic connections of PKCα-ir bipolar cells with photoreceptors in *M*. *domestica* (A-E) and *S*. *crassicaudata* retinae (F).** (**A**) Fluorescence double-labeling for PKCα (green) and the synaptic ribbon marker CtBP2 (red). In the OPL, PKCα-ir bipolar cell dendrites terminate in close proximity to the single-ribbon rod spherules (two marked by white arrows), but commonly not to the multi-ribbon cone pedicles (yellow arrow). (**B**) Electron micrograph of rod spherules (RS) in the OPL, where PKCα-ir dendrites (black staining) form invaginating contacts with the rod spherules (arrowheads). However, we did not observe any PKCα-ir dendrites in close proximity to the ribbon band of rod spherules (arrows). Nuc, rod nuclei in the ONL. (**C-E**) Double-labeling for PKCα (green) and CtBP2 (red) in flatmounted retina, where the focus is on the OPL. (**C**) The CtBP2 label shows the larger single ribbons of rod spherules and the somewhat smaller, clustered ribbons of two cone pedicles (positions marked in D). (**D**) Dense plexus of PKCα-ir dendrites, mostly sparing the position of the cone pedicles (circles). However, one dendrite ends at a cone pedicle (arrowhead). (**E**) The merge of (C, D) shows this dendritic tip in close proximity to cone ribbons, suggesting synaptic contact. Many of the other dendrites terminate close to rod ribbons. (**F**) Double-labeling for PKCα (green) and CtBP2 (red) in a *S*. *crassicaudata* retinal section, showing the PKCα-ir dendrites in close proximity to the single-ribbon rod spherules (three marked by white arrows), but not to the multi-ribbon cone pedicles (four marked by yellow arrows).

To elucidate whether the *M*. *domestica* PKCα-ir bipolar cells indeed are RBCs, we performed immunofluorescence double-labeling for PKCα and the synaptic ribbon marker C-terminal binding protein 2 (CtBP2), as well as pre-embedding immunoelectron microscopy (EM). This revealed invaginating contacts of PKCα-ir dendrites with rod spherules in the OPL (Fig 4A and 4B). In the EM material, we examined 89 rod spherules in series containing, on average, nine sections of 60 nm each, i.e. covering on average 36% of the spherule volumes (spherule diameter is about 1.65 μm). Notably, in none of these spherules did we observe any of the invaginating PKCα-ir dendritic tips in close proximity to the ribbon bands of rod spherules (Fig 4B), even when following the dendrites through the serial sections. Possibly this is due to a paucity of PKCα in these fine tips, insufficient sensitivity of our staining protocol and/or suboptimal tissue quality. Surprisingly, in the immunofluorescence double-labeled tissue we also found PKCα-ir dendritic endings at the base of some cone pedicles in close proximity to synaptic ribbons, suggesting sparse synaptic contacts with cones (example shown in Fig 4C–4E).

Double-labeling of *S*. *crassicaudata* retinal sections for PKCα and CtBP2 similarly showed a close proximity of the bipolar cell dendrites with rod synaptic ribbons in the OPL (Fig 4F). There was no evidence for contacts with the clustered ribbons of cone pedicles. However, these observations were limited to transverse sections and hence a small sample of cone pedicles. Thus, we cannot make a definitive statement about the presence or absence of potential cone contacts.

**Fig 5:**
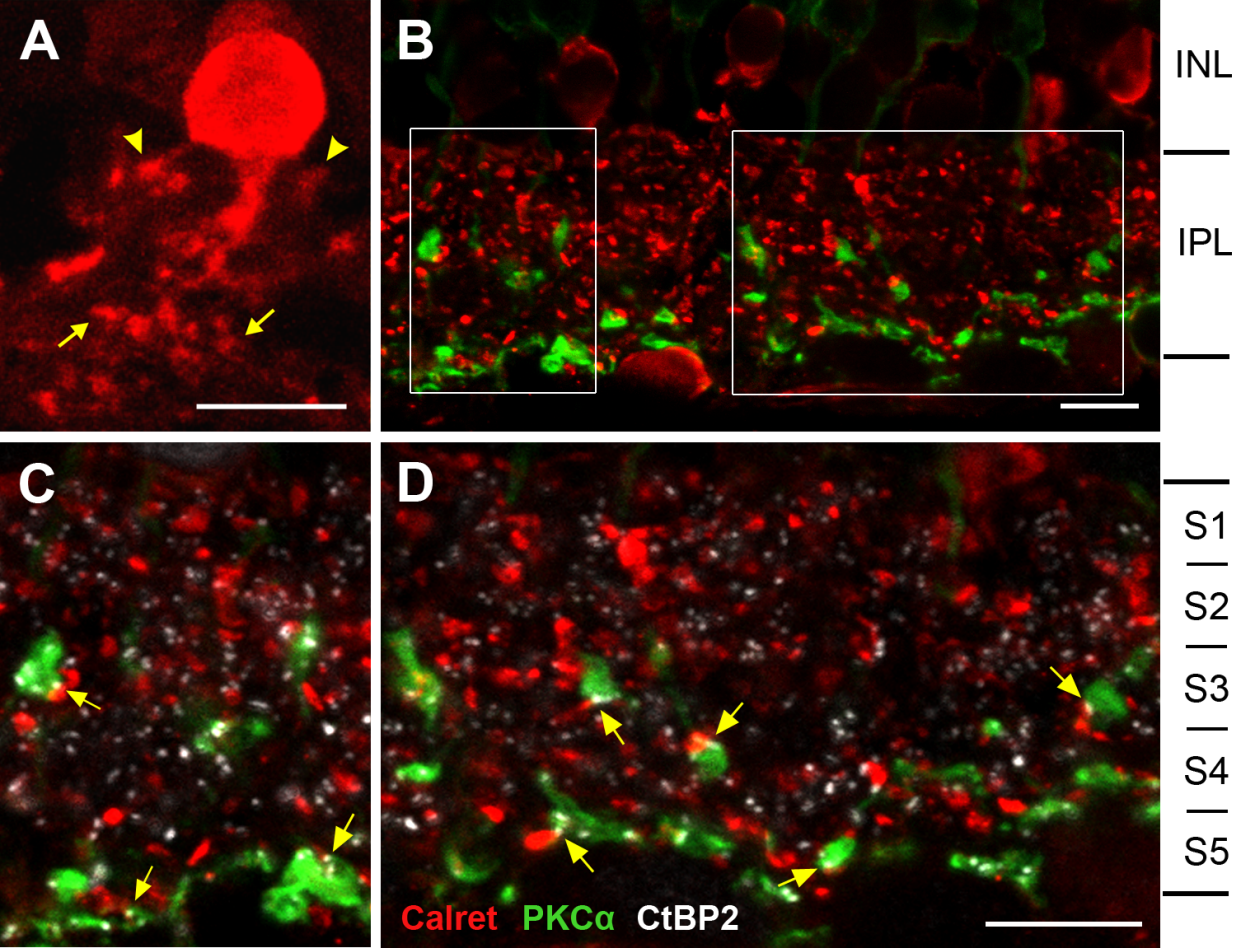
Synaptic connections of PKCα-ir RBCs with calretinin-ir AII cells. (**A**) Calretinin immunolabeling reveals amacrine cells with the typical bistratified AII morphology of processes in the outer IPL (arrow heads) and in the inner IPL (arrows). (**B-D**) Triple immuno-labeling for PKCα, calretinin and CtBP2. (**B**) Overview showing that RBC axon terminals and their *en passant* varicosities (green) are in close apposition to AII cell processes (red; CtBP2 label not displayed). (**C, D**) Magnified view of the two fields outlined in (B), showing all three labels. Labeled axon terminals have ribbon synapses, hence the CtBP2 label (white) at the contact points between RBCs and AII cells indicate that these are synaptic contacts (some marked by arrows). White CtBP2 puncta not associated with contact points between RBCs and AII cells are ribbon synapses of cone bipolar cells with ganglion cells or other amacrine cells. (B-D) is a single focus image from a confocal image stack. The IPL is divided into five equal strata S1 –S5. All scale bars are 10 μm.

The ‘classical’ rod signaling pathway in placental mammals is characterized by specific ribbon synapses between RBC axons and specialized amacrine cells, the AII cells, in the IPL. We performed triple immunostainings for PKCα, the AII cell marker calretinin, and CtBP2 to elucidate if this also holds for the marsupial *M*. *domestica* (Fig 5). Calretinin labeled a population of amacrine cells with the typical AII morphology known from placental mammals, having stouter processes with lobular appendages in the outer IPL and somewhat finer processes in the inner IPL (Fig 5A). The connections between RBC axons and AII cells in the IPL were analyzed in triple immunostained vertical sections. Examination of the single focal images of three confocal stacks (6–8 images per stack, z-axis distance 0.465 μm) from different retinal regions revealed a co-localization of all three markers at the PKCα-ir axon terminals and at the *en passant* varicosities (i.e., across strata S3 to S5), suggesting a specific functional connectivity between PKCα-ir RBCs and AII cells (Fig 5B–5D).

### Densities of rods, rod bipolar cells and AII amacrine cells

**Fig 6:**
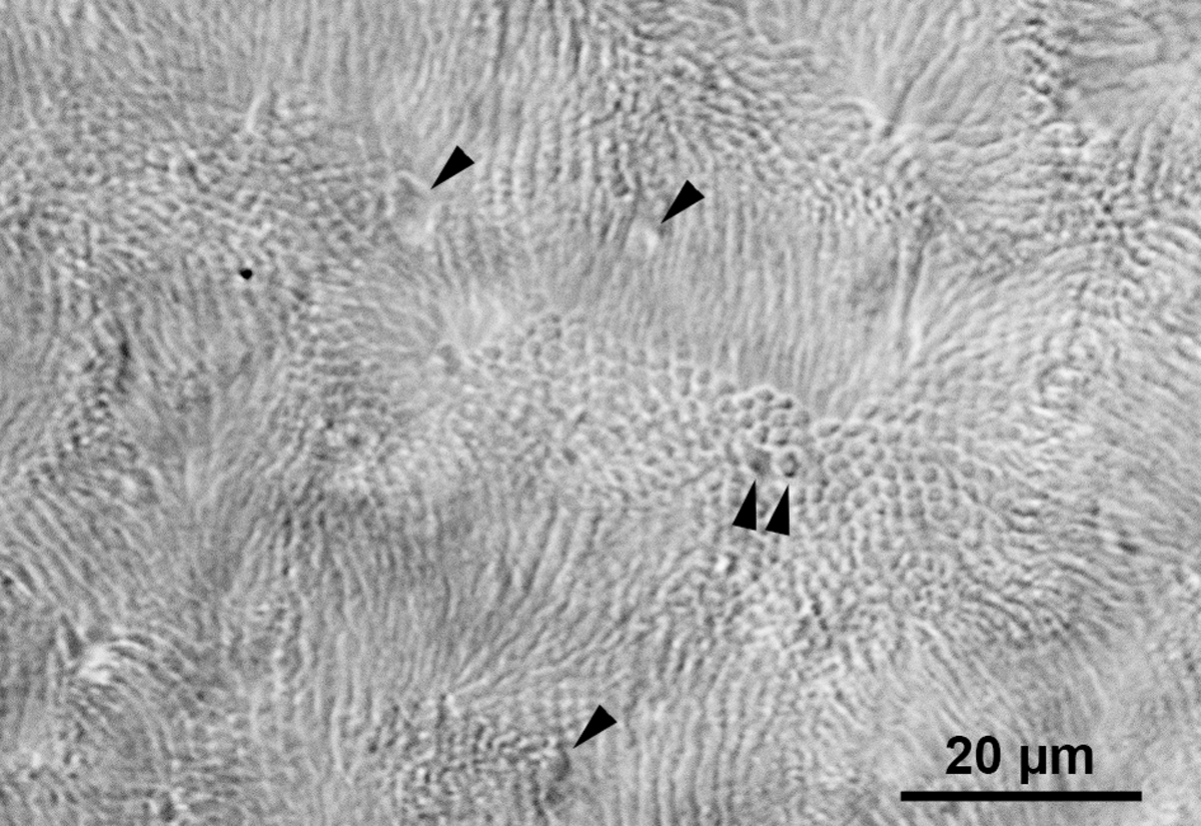
Flat view of the photoreceptor pattern in *M*. *domestica* retina. Differential interference contrast image (DIC) focused on the photoreceptor inner and outer segments in a flatmounted retina. The cobblestone-like mosaic of smaller profiles are the rods, the sparse larger profiles are the cones (some indicated by arrowheads). The twin arrowheads point to a double cone. As the photoreceptor outer segments were slightly squashed by the coverslip, they are bent sideways in patches, blurring the image. Rod density could be determined in the small patches where the rods are seen face-on. The retina was somewhat undulated, hence the focal plane of the micrograph grazes different levels of the photoreceptors, showing larger cone cross-sections in the upper when compared to the lower part of the image.

Rod densities ranged from 338,000 to 413,000 rods/mm² with a mean of 364,000 ± 22,000/mm² (± SD; n = 16), as assessed by DIC in several regions of the flatmounted retina md1 (Fig 6). There were considerable density differences between adjacent counting fields but no consistent density gradients across the retina. Rough cone density estimates indicated that the cones comprise 1% or less of the photoreceptors in *M*. *domestica*. The cone population contained double cones (marked in Fig 6), which are a common feature in marsupial retinae [6–8,32,33].

**Fig 7:**
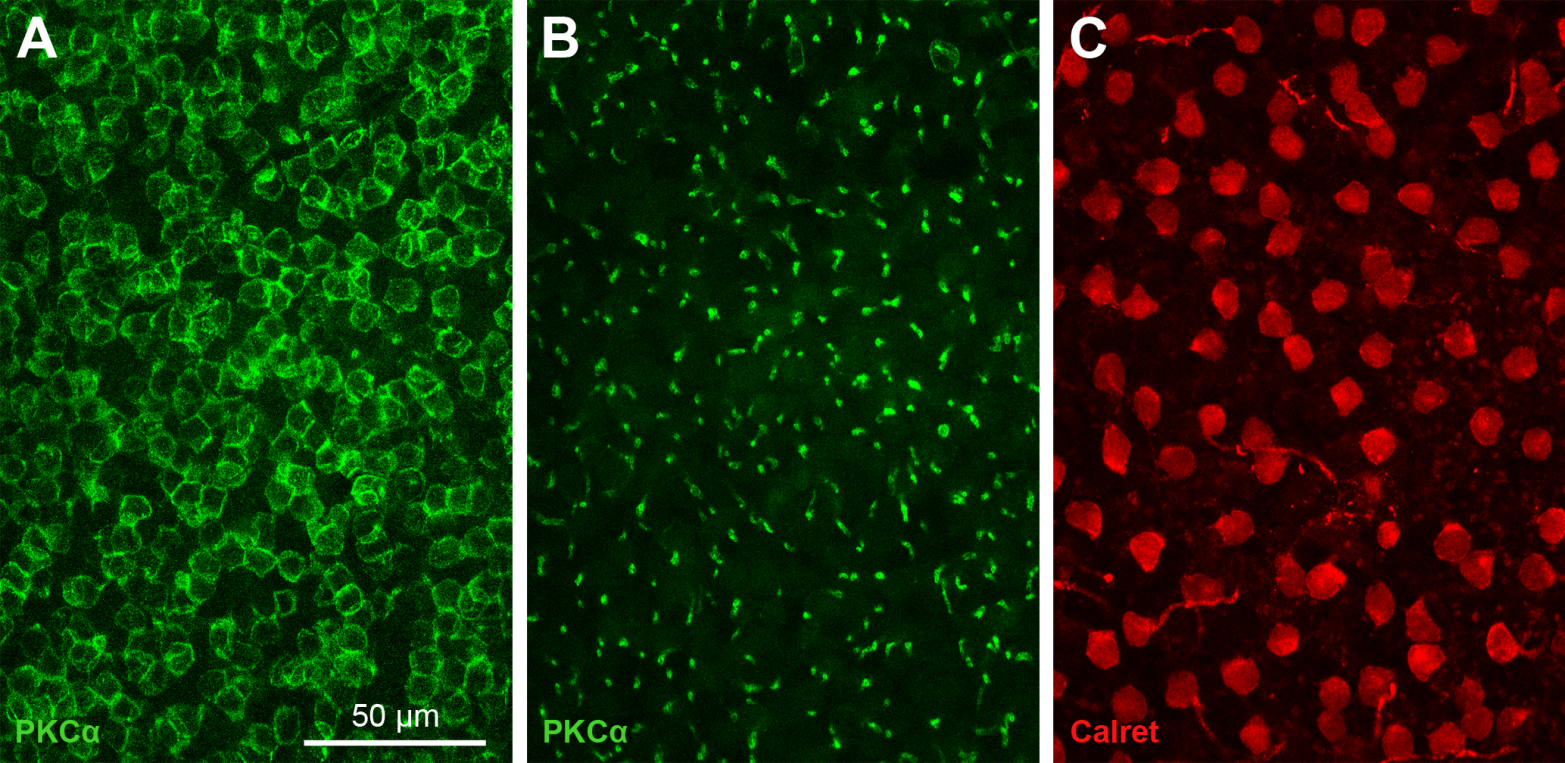
Flat view of RBC and AII cell populations in *M*. *domestica*. The images show one field in a retina double-labeled for PKCα and calretinin. (**A**) Focus on the densely packed RBC somata in the outer part of the INL; (**B**) focus on the vertically running RBC axons in the inner part of the INL; (**C**) focus on the AII somata in the inner part of the INL. Images are maximum intensity projections of confocal image stacks. The scale bar applies to (A-C).

**Fig 8:**
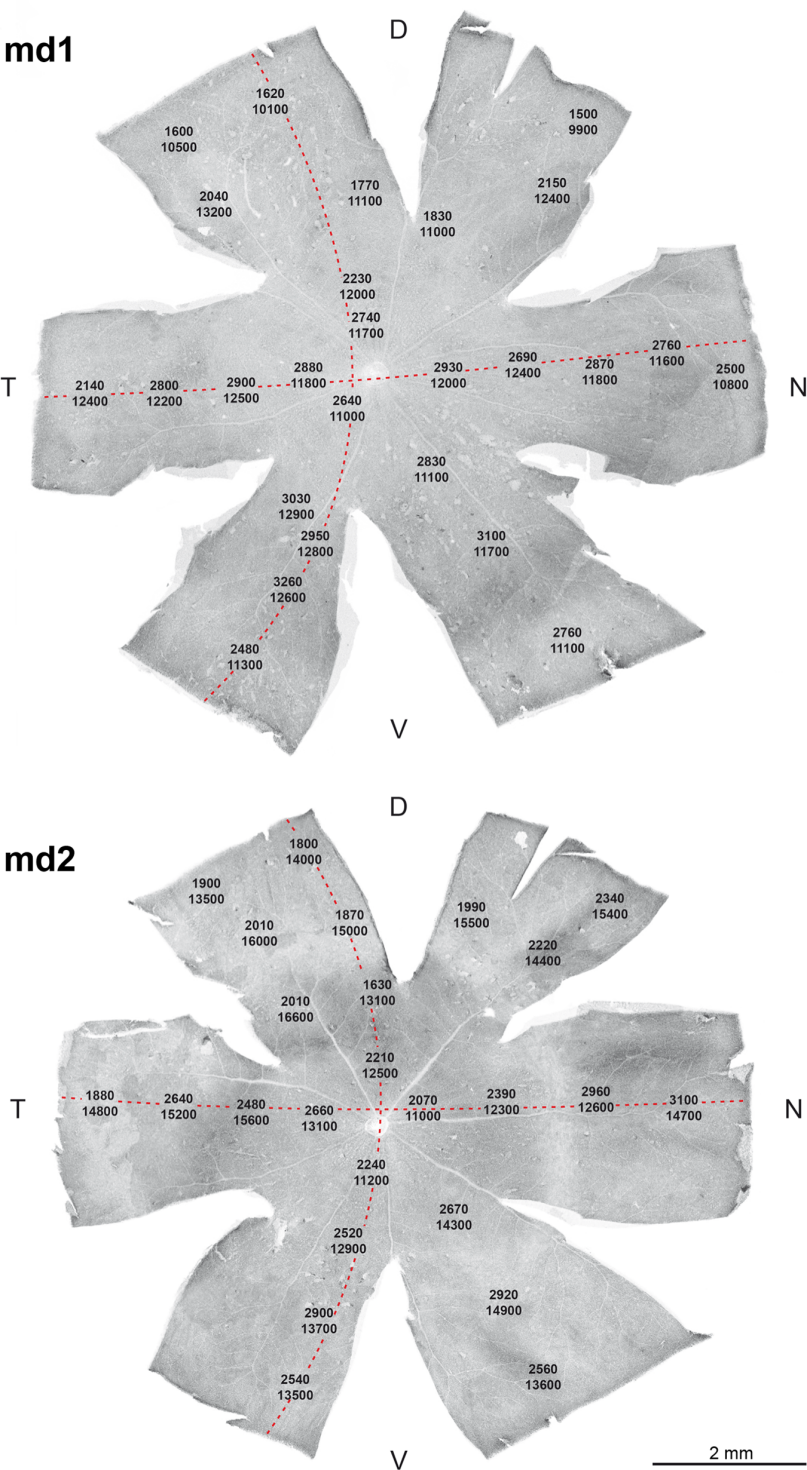
Density distributions of RBCs and AII cells in *M*. *domestica* retinae. For the two analyzed retinae md1 and md2, densities in the sampling fields were entered in photographs of the retinal flatmounts. Each number pair is positioned at the location of the sampling field, the top number is the AII cell density and the bottom number the RBC density in cells/mm². The broken red lines mark the temporal-nasal (T-N) and dorsal-ventral (D-V) transects along which the data of Fig 9 were sampled. The scale bar applies to both retinae.

Densities of RBCs and AII cells were assessed in two flatmounted retinae that had been double-labeled for PKCα and calretinin (Fig 7). The RBCs were counted at the level of their axons in the INL (Fig 7B) by following the axons through the confocal micrograph stack; this was more straightforward than counting the overlapping RBC somata in the outer part of the INL (Fig 7A). For the AII cells, somata were counted (Fig 7C). Cell densities found across the retinae are displayed in Fig 8. In retina md1, RBC densities ranged from 9,900/mm² to 13,200/mm² (mean ± SD: 11,685 ± 858/mm²), and AII cell densities ranged from 1,500/mm² to 3,260/mm² (2,499 ± 514/mm²). In retina md2, RBC densities ranged from 11,000/mm² to 16,600/mm² (13,969 ± 1,449/mm²), and AII densities ranged from 1,630/mm² to 3,100/mm² (2,340 ± 405/mm²). Hence, regional RBC densities varied by a factor of about 1.5, and regional AII cell densities by a factor of about 2. Furthermore, retina md2 showed generally higher RBC and AII densities than retina md1. This is unlikely to be an age effect as both animals were six months old.

For the *M*. *domestica* rod pathway, the mean cell densities in retina md1 gave an overall numerical convergence (density ratio) of 31.1 rods: 1 RBC, and of 145.6 rods: 4.7 RBCs: 1 AII cell. With the mean RBC and AII cell densities of retina md2 and the mean rod density of md1 (as no rod counts in md2 were possible), the numerical convergence in retina md2 was 26.0 rods: 1 RBC and 155.6 rods: 6.0 RBCs: 1 AII cell.

**Fig 9:**
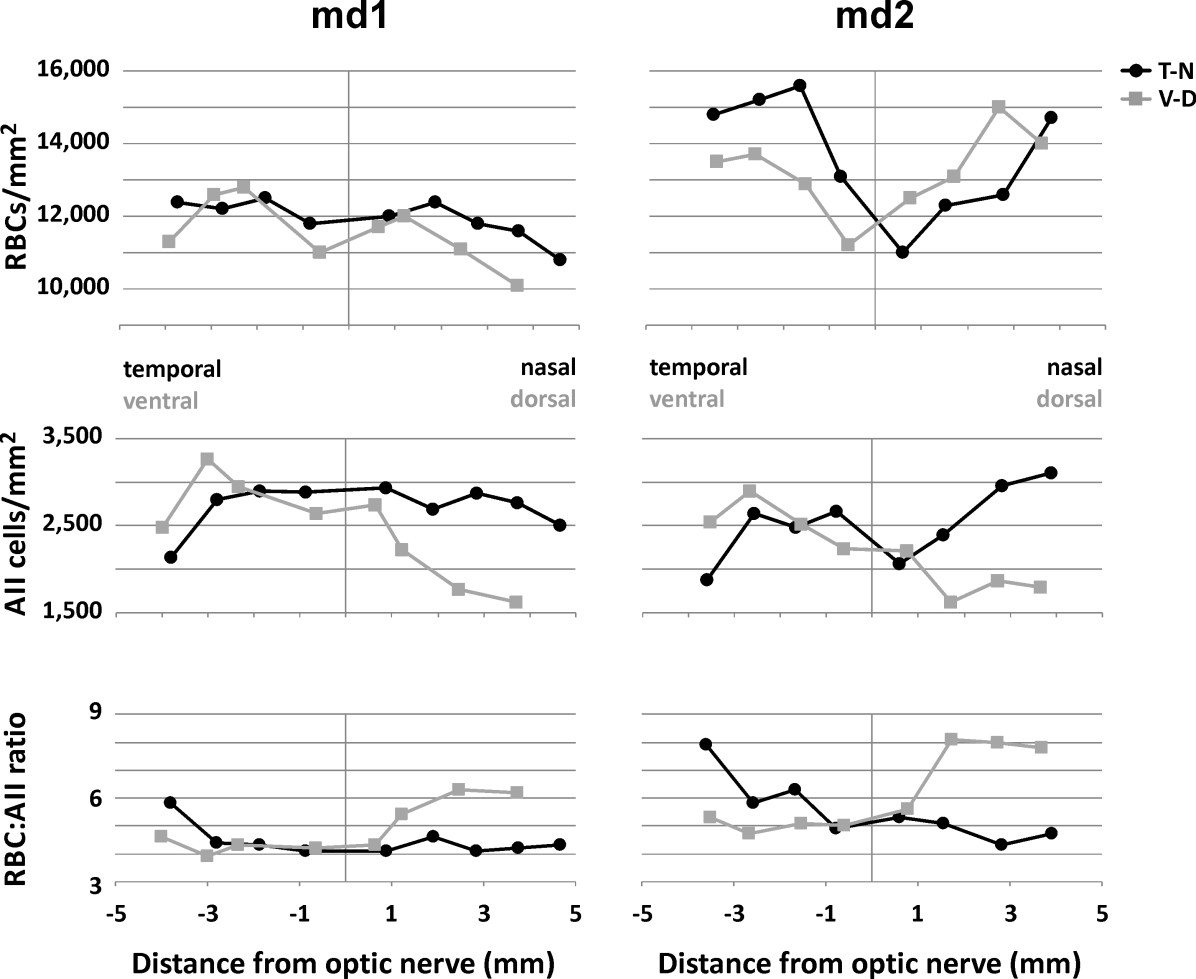
Gradients of RBC densities, AII densities and RBC/AII ratios across *M*. *domestica* retina. For retinae md1 and md2, the graphs show the density and ratio changes in relation to distance from the optic nerve head along the temporal-nasal (T-N) and dorsal-ventral (D-V) transects marked in Fig 8.

To better visualize density gradients across the retina, RBC and AII densities are also displayed in graphs, together with the resulting RBC/AII ratios (Fig 9). Density data in the graphs are taken along the temporal-nasal and dorsal-ventral transects indicated by the red lines in Fig 8. In retina md1, there was a shallow density decline of RBCs and AII cells from central to peripheral retina, with an indication of higher densities in the ventral retina compared to the dorsal retina, and a shallow central dip in RBC density. The RBC/AII ratio was relatively constant at 4–5 in central retina and nasal and ventral periphery, rising to around 6 in temporal and dorsal periphery. In retina md2, RBC densities were overall higher and showed an inverse gradient with lower densities in the central retina compared to the peripheral retina. Here, there was an indication of higher densities in the temporal retina compared to the nasal midperiphery. AII cell densities, on the other hand, were more similar to those in retina md1, again with higher densities in ventral retina, but also in nasal retina. The RBC/AII ratio was overall higher than in md1, being around 5 in the central retina and nasal and ventral periphery, and rising up to 8 in the temporal and dorsal periphery.

## Discussion

This study demonstrates that the retinal rod pathway in the gray short-tailed opossum *Monodelphis domestica*, a South American marsupial, closely resembles the well-known rod pathway in placental mammals [1,13,22]. The general retinal morphology of *M*. *domestica* is very similar to that of other nocturnal marsupials in that they possess a highly rod-dominated retina. The *M*. *domestica* rod densities of 338,000–413,000/mm² (mean 364,000/mm²) are in the range of those of the nocturnal American opossums *Didelphis virginiana* (310,000–485,000 rods/mm²; [5]) and *Didelphis aurita* (200,000–500,000/mm²; [6]), lower than those of the American mouse opossum *Thylamys elegans* (440,000–590,000/mm²; [7]) and of the house mouse (average 437,000/mm²; [34]), but higher than those of the nocturnal Australian brushtail possum *Trichosurus vulpecula* (199,000–288,000/mm²; [8]). The multi-tiered ONL and the peculiar heterochromatin arrangement in the rod nuclei (inverted nuclear architecture) are hallmarks of a ‘nocturnal’ retina [31].

On the other hand, cones are present at low densities. Our estimate of around 1% cones is comparable to similarly low cone proportions in other nocturnal marsupials (0.8–2% in *Didelphis virginiana*, [5]; ~1% in *Didelphis aurita*, [6]; 0.4–1.2% in *Thylamys elegans*, [7]; 0.5–1.1% in *Trichosurus vulpecula*, [8]). Our immunolabeling for the middle-to-longwave-sensitive LWS and shortwave-sensitive SWS1 opsins shows that these two genetically identified cone opsins [12] are indeed expressed in two separate cone populations, and that the SWS1 cones form a minority. The two cone types are the prerequisite for dichromatic color vision; the *M*. *domestica* SWS1 opsin is tuned to UV rather than blue/violet [12]. All three American marsupials for which the opsins have been analyzed are cone dichromats with LWS and SWS1 cone opsins [7,12]. Among Australian marsupials, both dichromatic and trichromatic species are found, with an, as yet, unidentified middle-wavelength-sensitive third cone opsin (summarized in: [11,35,36]). The fat-tailed dunnart *S*. *crassicaudata* belongs to the trichromats [9,11].

We provide clear evidence for the presence of marsupial RBCs, as the PKCα-ir bipolar cells form invaginating contacts with rod spherules in the OPL as well as ribbon synapses with AII cells in the IPL. Furthermore, their morphology and position within the retina, featuring a soma in the outer part of the INL and axon terminations in the innermost IPL, are in agreement with the RBC properties found in placental mammals ([18], which is reviewed in [13]). This suggests that the rod signaling pathway is conserved across the two clades of marsupials and placental mammals. PKCα immunolabeling is highly specific in identifying RBCs across placental mammals [18–20,22,37–40]. PKCα-ir bipolar cells have been reported previously in *M*. *domestica* [41] and in an Australian marsupial, the brushtail possum [37], but have not been shown to be RBCs. Our data are the first to establish RBCs in a marsupial. Amacrine cells with the narrow-field, bistratified morphology and calretinin and parvalbumin immunoreactivity typical of eutherian AII cells have been identified in *M*. *domestica* [42]. An amacrine cell type with AII-like morphology also has been described from Golgi staining in the Australian tammar wallaby *Macropus eugenii* [43]. In the current study we show the integration of these bipolar and amacrine cells in the rod signaling pathway.

**Fig 10:**
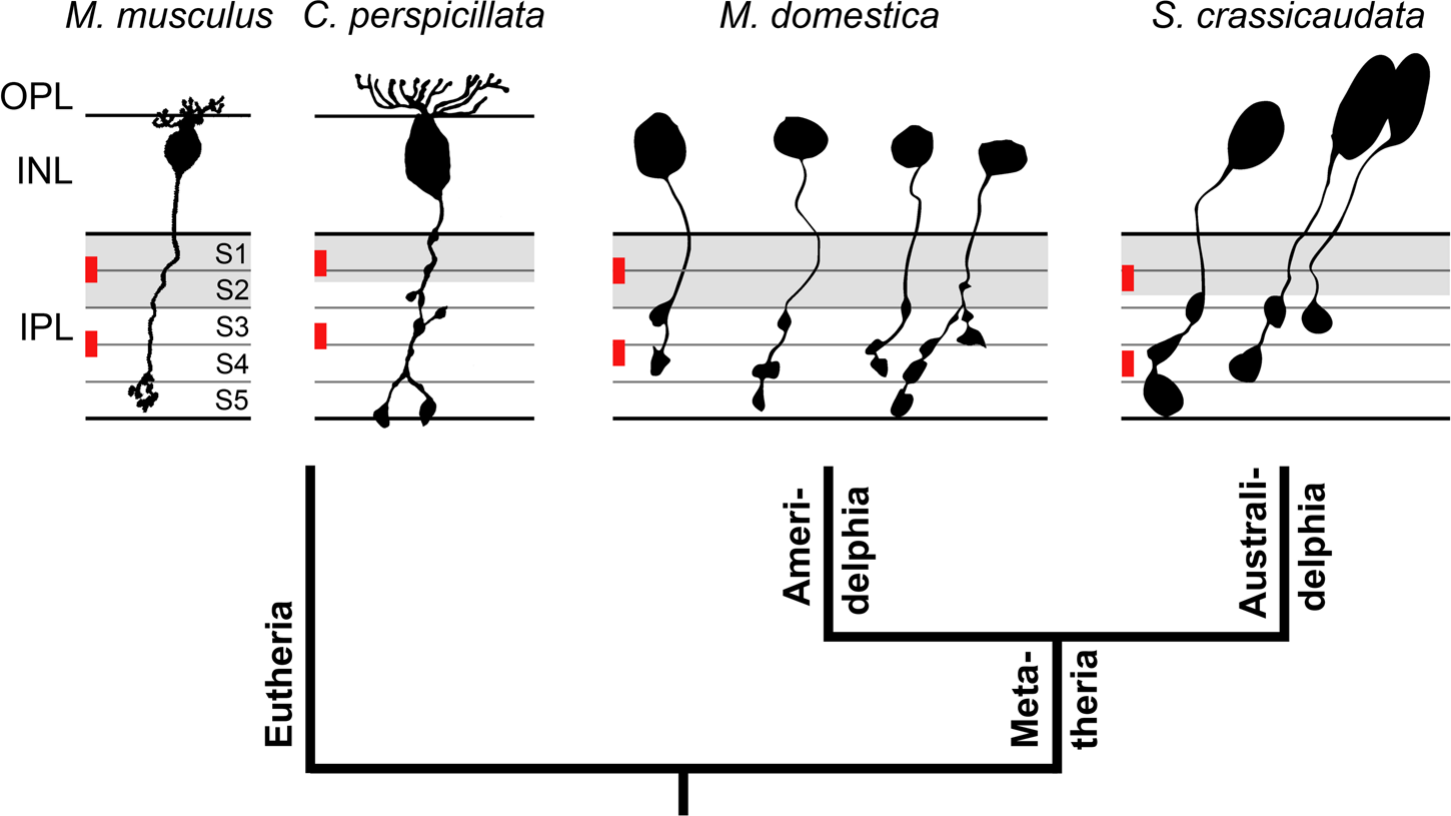
Comparison of mammalian RBC morphologies. Drawings of RBCs of the gray short-tailed opossum *Monodelphis domestica* and the fat-tailed dunnart *Sminthopsis crassicaudata* (PKCα immunolabel, present study), of the house mouse *Mus musculus* (dye-injected cell, modified from Fig 8 in [45]), and of Seba's short-tailed bat *Carollia perspicillata* (dye-injected cell, modified from Fig 7 in [40]). The gray lines divide the IPL into five equal strata S1 –S5. In *Monodelphis* and mouse, the OFF (outer) sublayer of the IPL is made up of the top two strata S1 and S2 (gray shading), the ON (inner) sublayer is made up of strata S3–S5. In *Sminthopsis*, the OFF sublayer includes S1 and a large part of S2, and in *Carollia*, it includes S1 and only a small part of S2. The positions of the outer (OFF) and the inner (ON) band of cholinergic amacrine cell processes (ChAT bands) are indicated in red. Drawings are scaled to equal IPL thickness.

Interestingly, the RBCs of *M*. *domestica* often have axonal side branches and *en passant* varicosities at more outer IPL levels than stratum S5, and these appear to also form synapses with AII cells. The presence of similar multistratified PKCα-ir RBCs in the fat-tailed dunnart *S*. *crassicaudata* shows that this feature is shared by both an American and an Australian marsupial, suggesting it may be a homologous feature of the marsupial retina. We have observed multistratified PKCα-ir bipolar cells in further American (*Thylamys elegans*) and Australian (*Pseudocheirus peregrinus* and *Trichosurus vulpecula*) marsupials, but we have also observed monostratified, i.e. conventional, PKCα-ir bipolar cells in some other Australian marsupials (*Isoodon macrourus*, *Pseudocheirus occidentalis*, *Macropus giganteus*, *Vombatus ursinus*, *Phascolarctos cinereus*), revealing that multistratification is not ubiquitous across the two groups (unpublished observations). Actually, Young and Vaney [37] report the PKCα-ir bipolar cells of the brushtail possum (*Trichosurus vulpecula*) to be monostratified, whereas our material shows them as bi- or multistratified. The limited material presently available does not allow us to resolve this discrepancy but perhaps there is RBC stratification heterogeneity in this species. Multistratified RBCs seem rare across placental mammals, but have been demonstrated to exist in microchiropteran bats [40] and in elephants [44]. Fig 10 compares the morphology of *M*. *domestica* and *S*. *crassicaudata* RBCs to those of the house mouse (*Mus musculus*) and a microchiropteran bat (*Carollia perspicillata*). Similarities between the species include the soma position in the outer part of the ONL, multiple fine dendrites contacting the rods in the OPL, and the major axon terminal endings in the innermost part of the IPL (stratum S5). Differences include the presence of additional axon terminals and varicosities in more outer IPL strata in the two marsupials and the bat, giving these RBCs a bistratified or diffusely stratified appearance. Notably, these additional synaptic output sites are all located within the presumed ON sublayer of the IPL, as indicated by the labeling for Gγ13 [23,40,45]. In the mouse retina, Gγ13 is considered to be a G protein subunit specifically participating in the signal transduction by ON bipolar cells [23]. The parsimonious assumption is that such molecular specificity is evolutionarily preserved across placental and marsupial mammals.

One unexpected feature of the *M*. *domestica* RBCs are their potential contacts with cones, indicated by the close proximity of some PKCα-ir dendrites to cone pedicles. Unfortunately, the tissue quality of the EM sections was not good enough to firmly establish whether such bipolar cell dendrites formed actual synapses with the cone pedicles, or whether they just passed close by. Cone contacts of RBCs go against the classical view of a clear dichotomy of rod and cone bipolar cells in mammals. The presence of such contacts might suggest that the marsupial rod pathway is evolutionarily intermediate between the unspecific rod- and cone-connecting bipolar cells of”lower” vertebrates like zebrafish [46] and turtles [47], and the rod-specific and cone-specific bipolar cells in placental mammals. However, recent ultrastructural serial reconstructions of 141 mouse RBCs showed that 75% of them also made contact with one or several cones [48]. In the baboon retina, 12% of the RBCs were found to also contact cones [49]. Hence, mixed rod and cone input to RBCs would not constitute a difference between placental and marsupial mammals. We report our preliminary observations to stimulate further study of this aspect.

The *M*. *domestica* RBC density of 9,900–16,600/mm² (range in two retinae) is close to that of the house mouse (15,000/mm², [50]), markedly lower than that of the cat (30,000/mm², [51]), but markedly higher than that of the rabbit (3,500–7,000/mm², [20]). Interestingly, the RBC density range of the macaque monkey (5,000–20,000/mm², [38]) comprises densities that are unexpectedly high for a diurnal mammal. No RBC density values are available for other marsupials.

The *M*. *domestica* AII cell density of 1,500–3,260/mm² is also in the range found among nocturnal to crepuscular placental mammals; for example the house mouse has 3,000–4,000 AII cells/mm² [52], the rat has 2,000–7,000 AII cells/mm² [53], the cat has 500–5,300 AII cells/mm² [54], and the rabbit has 400–3,000 AII cells/mm² [55,56]. Interestingly, the diurnal macaque monkey with 800–5,000 AII cells/mm² [57] does not differ substantially from the nocturnal-to-crepuscular cat and rabbit. In all these species, the highest AII cell densities are found in central retina (with a local minimum in the rodless fovea of the macaque), and the lowest densities are found in the dorsal peripheral retina. *Monodelphis domestica* also has the lowest AII cell densities in dorsal retina, whereas its higher AII cell densities in ventral mid-peripheral retina have no equivalent in the studied placental mammals.

The numerical convergence (density ratio) in the *M*. *domestica* rod pathway of 145.6–155.6 rods: 4.7–6.0 RBCs: 1 AII cell compares to a ratio of 110: 7.3: 1 in central cat retina [51], ~100: 2: 1 in the visual streak of the rabbit retina [20,56], and ~60: 4: 1 in the midperipheral macaque retina (combined from [38,57]). The *M*. *domestica* ratio of 26.0–31.1 rods: 1 RBC compares to a very similar 29: 1 ratio in the mouse retina [34,50], to a 15: 1 ratio in the central cat retina [51], to a 43–58: 1 ratio across the rabbit retina [20], and to a 5–20: 1 ratio across the macaque monkey retina [38]. Hence, the opossum ratio is within the broad range found among placental mammals.

In summary, our data demonstrate the presence of multistratified RBCs in rod-dominated marsupial retinae, and their integration in the rod signaling pathway, comprising invaginating contacts with rod spherules, and ribbon synapses with AII cells. Interestingly, we also found potential contacts of RBCs with cone pedicles. Although RBC multistratification is rare in placental mammals and RBC contacts with cones have so far only been demonstrated in mouse and baboon, our findings conform to the general rod signaling pathway seen in placental mammals. Rod, RBC and AII cell densities as well as their density ratios were found to be within the broad range present across placental mammals. Our data add to the sparse literature on scotopic vision in marsupials. The similarity of the rod pathway in marsupials and placental mammals suggests a common evolutionary origin of this pathway.
